# Stigma and mental health problems in an Indian context. Perceptions of people with mental disorders in urban, rural and tribal areas of Kerala

**DOI:** 10.1177/00207640221091187

**Published:** 2022-05-13

**Authors:** Raghu Raghavan, Brian Brown, Francesca Horne, Sanjana Kumar, Uma Parameswaran, Ameer B Ali, Ardra Raghu, Amanda Wilson, Nadia Svirydzenka, Chitra Venkateswaran, Manoj Kumar, Sreedevi R Kamal, Andy Barrett, Chandra Dasan, Aarcha Varma, Asha Banu

**Affiliations:** 1 De Montfort University, Leicester, UK; 2 Mental Health Care and Research Foundation, Kochi, Kerala, India; 3 Mental Health Action Trust, Kozhikode, Kerala, India; 4 Excavate Theatre, Nottingham, UK; 5 Lokadharmi Theatre, Kochi, Kerala, India; 6 Tata Institute of Social Sciences, Mumbai, India

**Keywords:** Mental health, stigma, India, community

## Abstract

**Background:**

The concept of stigma has been widely used to understand patterns of discrimination and negative ideas surrounding people with mental health problems, yet we know little of the specific nuances of how this might operate beyond the ‘Global North’.

**Aim:**

This paper aims to explore the notion of stigma in an Indian context by considering the lived experience of patients, carers and community members.

**Methods:**

A sample of 204 participants, representing mental health patients, informal carers and community members was recruited from urban and rural areas in Kerala, India. Participants took part in interviews where they were encouraged to talk about their experiences of mental ill health, attitudes towards these problems, barriers encountered and sources of support.

**Results:**

Experiences akin to the experience of stigma in Europe and the United States were elicited but there were important local dimensions specific to the Indian context. The difficulties faced by people with diagnoses of mental disorders in finding marriage partners was seen as an important problem, leading to marriage proposals being refused in some cases, and secrecy on the part of those with mental health problems. Rather than the ‘self-stigma’ identified in the US, participants were more likely to see this as a collective problem in that it could reflect badly on the family group as a whole rather than just the sufferer.

**Conclusions:**

In the Indian context, the idioms of stigma emphasised impairments in marriage eligibility and the implications for the family group rather than just the self.

## Introduction

According to the World Health Organisation (WHO), it is estimated that globally 450 million people suffer from mental disorders (WHO, 2012). Around 80% of these live in low-and middle-income countries (LMICs). Despite this, little is known about how these nations compare to higher income countries with a greater healthcare resources. This paper will explore the perceptions and beliefs of people with mental health problems, informal carers, and community members concerning mental ill health across four different sites in Kerala, India, namely Ernakulum, Palakkad, Calicut and Malappuram. Despite India’s increasing literacy rates and economic growth 197.3 million people in 2017 experienced mental health problems (India State-Level Disease Burden Initiative Mental Disorders Collaborators, 2019). There are concerns that people with mental health problems suffer discrimination and exclusion as well as negative typifications and expectations – what in the Global North is termed ‘stigma’ – which can lead to not seeking adequate care, treatment or support (Venkatesh et al., 2015).

Scholars in the USA have, broadly speaking, defined two different types of stigma: societal and self-stigma. Societal stigma is defined as the ‘disproval of, or discrimination against a person based on perceivable social characteristics that serve to distinguish them from other members of a society’ (Goffman, 1963). Self-stigma is defined as ‘persons with mental illness, living in a culture steeped in stigmatising images, may accept these notions and suffer diminished self-esteem and self-efficacy as a result’ (Corrigan & Watson, 2002: 35). In the North American and Western European experience, a combination of these stigmas is conjectured to lead to severe feelings of isolation, as the attached stigma on individuals makes them prone to severe consequences such as substantial loss of self-esteem, self-stigmatisation, reduced job opportunities and social exclusion (Hall et al., 2019).

As a way of encouraging more compassionate and supportive attitudes towards people with mental health problems, the promotion of mental health literacy (MHL) has been advocated (Altweck et al., 2015). Jorm et al. (1997, p.184) defined MHL as ‘knowledge and beliefs about mental disorders which aid their recognition, management or prevention’. In attempting to promote MHL many authors recommend similar programmes, either in the forms of spreading awareness about the bio-medical cause of mental illness or anti-stigma campaigns (Gaiha et al., 2014). These may work in many westernised countries, however, in a country like India, where religion, spirituality and family hierarchies play a major role in daily life, these regimes are less effective (Svensson & Hansson, 2016). Moreover, mental health initiatives originating in the global north may have limited potential in low-and-middle income countries (Mills & Hilberg, 2019) as they often promote a medico-psychotherapeutic position with regard to mental health which is at odds with local beliefs and practices.

Over the past decade, some research suggests a divide in MHL rates between people living in rural and urban areas (Kishore et al., 2011; Murthy et al., 2020; Poreddi et al., 2015). Yet Ogorchukwu et al. (2016) indicate the urban/rural divide is not as wide as first thought and that it is uncertain whether there is a tangible difference in perceptions of mental health between rural and urban areas. Accordingly, this paper will explore commonalities and differences in perceptions of mental health difficulties across rural, urban and tribal areas of Kerala, India. Additionally, we were concerned to examine the extent to which the construct of stigma, developed in the Global North, could usefully illuminate the beliefs, experiences and practices described to us. This has implications for how health educators and campaigners in the future might seek to tackle the social barriers experienced by people with mental health problems and their families, as well as contribute to the cross-cultural interrogation of concepts such as stigma through which mental health problems are understood.

## Aims and methodology of the study

The study reported here formed part of a larger project on mental health literacy in India funded by the UK Research Councils, to explore the role of applied theatre and creative arts in enhancing mental health literacy. The larger project involved a multidisciplinary team comprising mental health specialists, cross cultural scholars and dramatists and used narrative interviews with local people to help design plays and films which were then used to prompt further discussion. The aspect of the project reported here draws upon 204 interviews, exploring how patterns of social exclusion and barriers to participation – ‘stigma’, in other words – were manifest and how this might parallel or differ from existing conceptions in the literature. Secondly the study aimed to shed light on whether and to what extent differences were visible between urban, rural and tribal areas.

The study took place in Kerala, a southern state in India with the highest Human Development Index (HDI) of 0.79 in the country. The state has the highest literacy rates among all Indian states at 98.9% and a life expectancy of 74 years which is among the highest in the country. The majority of the population are of the Hindu faith with significant Muslim and Christian minorities. Despite the health advances in Kerala, 14.4% of population aged 18 and above experience mental ill-health once in their lifetime, with 12.5% of individuals with suicidal risk. This is the highest of all the states of India and is almost twice the national rate of 6.4% (National Institute of mental Health and Neurosciences, 2017). This elevated rate is attributed to the high number of people suffering from depression in the state (Shaji et al., 2017). Mental health experts link the rise of depression to Kerala’s fast socioeconomic transformations (Halliburton, 2009). These have coincided with decline of the joint family system, discrepancy between high standards of education and low employment, and labour migration to the Gulf States, resulting in ‘gulf depression’ of these migrants and of the women left behind. Moreover, the gap between high expectations, and often harsh socioeconomic realities, the heavy consumption of alcohol, and pressure exerted on children by the school system, also reportedly contribute to depression (Halliburton, 2009).

The sample was constructed to include four different districts that consist of villages with farming communities, tribal colonies with low-income levels and living standards and towns and cities with higher income and living standards. The participants were recruited from eight different locations across Kerala, including Rural and Urban areas; Ernakulam, Chottanikkara (Rural) and Edappally (Urban); Palakkad (Rural and Tribal); Malappuram (Urban and Rural); Calicut (Urban and Rural). The Ernakulam district is the fastest developing region in Kerala gaining national and international level importance in business, trade, technology, education, health and tourism. Edapally, the urban site is a hub of leading business centres, educational institutions, health care centres as well as religious institutions. Chottanikkara, the rural site is located 16 kms from the main town and is home to the famous Chottanikkara temple. In Palakkad district, Elappully is a rural area near the Kerala–Tamil Nadu border with a mix of Tamil Nadu and Kerala cultures. The second site in Palakkad, Attappadi, is a hilly area with reserved forests. It is one of the least developed and least literate places in Kerala. The majority of the population in Attappadi are from tribal communities. Calicut the third district is also an economical hub of Kerala with centuries of history in trade through the Indian Ocean. Payyoli is a rural coastal area situated 36 kms from the central town of Calicut. Malappuram is the third-largest district of Kerala by area and the third biggest contributor to the GDP of Kerala. Over 70% of the population are of Islamic faith. Ponnani, the urban area is a major fishing centre. Vailathur is a rural village area located on the border of Malappuram and Thrissur districts.

Across all locations, an approximately comparable number of females (116) and males (88) participated. Table 1 provides a breakdown of participants by status, location and age.

**Table 1.** Breakdown of interview participants by location.

Location	Urban vs rural	Patients number interviewed (N), mean age and standard deviation	Caregivers number interviewed (N), mean age and standard deviation	Community members number interviewed (N), mean age and standard deviation
Ernakulam	Rural (Chottinikkara)	N = 7 Age = 42.1 (s.d. 4.01)	N = 8 Age = 50.8 (s.d. 16.8)	N = 5 Age = 40 (s.d. 11.4)
	Urban (Edappally)	N = 8 Age = 32.2 (s.d. 14.1)	N = 8 Age = 50.8 (s.d. 14.9)	N = 8 Age = 34 (s.d. 12.3)
Palakkad	Rural	N = 8 Age = 39.5 (s.d. 7.5)	N = 8 Age = 44.5 (s.d. 18.9)	N = 8 Age = 40.6 (s.d. 14.9)
	Urban	N = 8 Age = 41.3 (s.d. 17.1)	N = 9 Age = 53.1 (s.d. 17.7)	N= 8 Age = 60.7 (s.d. 9.9)
Malappuram	Rural	N = 8 Age = 46.8 (s.d.11.6)	N = 7 Age = 56 (s.d. 10.6)	N = 7 Age = 41.3 (s.d. 5.8)
	Urban	N = 8 Age = 46.3 (s.d. 9.6)	N = 8 Age = 42.8 (s.d. 15.8)	N = 7 Age = 49.5 (s.d. 11.1)
Calicut	Rural	N = 8 Age = 42.7 (s.d. 10.5)	N = 7 Age = 60.4 (s.d. 10.8)	N = 7 Age = 46.3 (s.d. 13.9)
	Urban	N = 7 Age = 33 (s.d. 8.1)	N = 8 Age = 46.6 (s.d. 1.7)	N = 5 Age = 30.8 (s.d. 13.3)

Following ethics committee approval in the researchers’ host institution, and informed consent from participants, the interviews were undertaken using a guide which focussed on onset of symptoms, course, significant events in the lives of the patients, explanation for perceived abnormal behaviour and experiences with treatment. The interviews were carried out in appropriate local languages, by researchers trained to undertake qualitative interviews. The interviews were recorded, translated and transcribed. Participants provided detailed narratives that were then analysed for themes relating to sources of sigma, adverse responses from others and resources of resilience used to address the risk, hardship and adversity encountered as a result of mental ill health.

## Findings

Whilst there were aspects of the experience of mental ill health that could be seen in terms of the notion of stigma as it has been presented in the literature so far, there were several dimensions which were distinctive to the Indian context, which will be the focus of our presentation below.

### Societal stigma: Marriage prospects

An important dimension of the experience of mental ill-health concerned the issue of marriage. Whilst mental health and romantic relationships have been touched upon in European and north American literature (e.g., Brown, 2020) a striking aspect of stigma which was distinctive to our data was the pre-eminence of concerns about marriage and marriage prospects.

Despite the introduction of the Mental Health Act 2017 in India, which aimed to reduce the overall social stigma associated with mental illness, there is still a strong sense that the marriage prospects of those who have a mental illness or family members are impaired. Marriage is an important aspect of a person’s life in India as it is seen as a necessity across all religions. Among the three major religious groups in the area marriage was seen as important. For Hindus, it is regarded as an essential ‘Samskara’ (sacrament for every Hindu) (Sharma et al., 2013); for Muslims, it is a fundamental building block of life (Jafaar-Mohammad & Lehmanm, 2011); and for Christians it is a gift from God. As marriage was considered imperative for most of the population, people who are not seen as ‘suitable’ partners can experience significant stress and discrimination.

People who suffer with mental illness can be determined as not ‘suitable’. People of all religions reported experiencing some discrimination regarding their marriage prospects. The following excerpt from a carer of a person diagnosed with depression in Chottinikkara demonstrates the societal stigma associated with the fear their loved one would not be able to get married due to adverse judgements by others.

‘He isn’t married. So, we don’t say all this to others fearing it might stop his marriage from taking place. Fearing people might start spreading the news that he is a guy with mental problems and defame him. It’s not because of anything else’ (Caregiver 32, Hindu, Rural Chottinikkara)

The view mental illness is hereditary was believed to cause issues in getting a marriage proposal. This was explained by a mill owner from urban Malappuram.

‘If some marriage proposals come if they talk in the society that he or she had some issues there can be problem in getting the proposal ahead. Otherwise, people will talk that hereditarily the boy or girl has some problems’. (Community Member 17, Male, Hindu, Urban Malappuram)

Most people who had commented on the damaging effect of stigmatised views of mental ill-health on marriage was women. The culturally dominant practice of arranged marriages where the family has power and control in choosing a mate for the son or daughter is thus challenged. If a man or woman’s parents do not approve of their relationship, they may be forced to marry someone else of whom the family approves. For example, one of the participants was worried that if the family of the daughter whom they wanted their son to marry were to find out he had a mental illness, they would not be approve of the marriage.

Patient 61, a female in her 20s, from Edapally poignantly explained how mental health stigma was a major fear among family members, especially when they were seeking to make a ‘partnership’ between two families. Patient 56, a female in her 40s, who experienced this first-hand from a potential partner after she disclosed her mental disorder to him:

‘I just told the truth that I am taking medicine and its only just for anxiety, I was trying to make him understand that it is not a big thing, but that person completely ruined it and he portrayed it as if I am not mentally fit to marry or something and he declined our proposal and left’. (Patient 56, Female, Hindu, Edappally)

Whilst the reluctance to marry someone with a history of mental health problems may be seen as an aspect of stigma, forming such a relationship commits the unaffected partner to a role as an informal carer in a context where professional support and resources are far more sparse than is customary in countries such as the UK (Sahithya & Reddy, 2018). Sustaining a marriage in these circumstances can be financially and emotionally demanding, both in supporting that person and paying for treatment. Imagining the future demands of the caring role may play a much of a role as the fear of judgement from society. It was explained by a community member in urban Calicut who was a PhD scholar, that a marriage proposal can get cancelled if the prospective bride or groom visits a psychiatrist.

‘We’ve been hearing since childhood that a psychiatrist is someone different. Even marriage proposals get cancelled if the bride or groom is a psychiatrist or the one who consulted a psychiatrist’. (Community Member 44, Male, Hindu, Calicut Urban)

In some families, we were told that family members of those who have mental illness often conceal this, until after the marriage has taken place as in case the prospective partner (or their family) would refuse the marriage if they found out. This was also described by a female community member in rural Malappuram, where a woman’s husband in her village was diagnosed with a mental illness before they got married, however it was concealed at the time of the marriage proposal by his family. She explained how difficult it was for the woman to readjust.

‘She got to know about her husband’s mental health status only after her marriage. And now, she is like “this is my fate, and I am accepting that.” She told that at time she feels to give up everything’ (Community member 29, Female, Muslim, Rural Malappuram)

The sense of loss in connection with marrying someone with mental health problems was a strong feature; it was seen as closing off many opportunities, both in terms of the demands of the caring role, and as we shall see in the section below in terms of wider social opportunities.

### Stigma and the perception of people with mental health problems

In line with understandings of stigma in Britain and the United States, participants described examples of stigmatised perceptions of people with mental ill-health, consistently across urban and rural areas in Palakkad, Ernakulum, Calicut and Malappuram. Patients from both urban and rural districts talked about their experiences with stigma among their communities. The following excerpt is from an interview with a patient in Chottanikkara which is illustrative of the discrimination she has faced after talking about her mental illness.

‘They consider that as a shame. When we tell others about the illness, they call it madness’ (Patient 49, Female, Hindu, Chottinikkara)

The term ‘shame’ attaches to the individual concerned and their family. She also feels that she is characterised with the pejorative term ‘madness’, too, which akin to shame itself has a persistent quality. The use of derogatory terms to describe people with mental illness was also seen in a patient from Edappally.

‘Brother called me as “mental girl” and it hurt me badly’ (Patient 61, Female, Hindu, Edappally)

By contrast another community member, who was a journalist, explained how she visited different regions across Chottanikkara and believed this stigma was dissipating. Participant 54, a community member from Edappally went on to talk about how education has improved her understanding of mental illness. She talked about how the media have influenced people’s beliefs around psychiatrists and mental health support which could have affected the perception help-seeking for mental illness. It has long been suggested by scholars of mental health in India that media portrayals can influence those who have been diagnosed with a mental disorder too (Padhy et al., 2014). The influence the media were believed to have was thought to affect many patients. This was elaborated upon by a community member, who was a journalist.

‘TV and media people see the locking up and shock treatment etc. Even though it is a physical aspect, the nature of it is not violent as we see in TV. We see that in cinemas that a person who doesn’t have mental issue is getting mental problem due to all these’. (Community Member 54, Edappally)

However, despite the evidence that some forms of stigma surrounding mental ill-health still exists in Indian society, patients in urban Edappally mentioned that they were very well supported by their community and close relatives and friends. The families who were supportive of the patients were associated with medical professions and thus educated in scientific conceptions of mental health.

‘Society means for me it’s like my friends and family. So, as I said like they were very supportive to me. So, with them, when I talked my problems to them, they used to listen it and they used to give me suggestions or support me and that makes me feel good’. (Patient 58, Female, Hindu, Edappally)

There were many patients who felt as if they were supported in across the different districts, in Calicut, for example, a female in her 30s, felt very supported by her husband.

‘Now I am married, and husband is very supportive(smiling)’. (Patient 33, Female, Rural Calicut).

Despite a number of patients across all districts claiming they had family support, in Palakkad especially many participants felt society could do more to support people with mental health problems. For example:

‘The lack of support and from patients and their family, not having medicines et cetera are the main issues. Due to the lack of support systems in the family two or three patients dropped out from here (respite centre in Palakkad)’. (Community Member 1, Male, Hindu, Urban Palakkad)

In this quotation, the support from clinics is seen as working in synergy with support in families and communities – if the latter is lacking, people are believed to be apt to drop out. Thus, there is an acknowledged role for informal carers, to whom we will turn in the next section.

### Lack of support from community for carers

Caregiver participants, especially in the rural Chottinikkara and Malappuram, talked about not receiving support from their friends and community and even felt as if they were ‘alienated’ from society. One participant who cares for his two children with mental health problems, mentioned how his brother-in-law had no support from his family and committed suicide.

‘Families need to be sensitive to people with such illness, my brother-in-law committed suicide because his family wasn’t supportive enough’ (Caregiver 28, Rural Malappuram)

Many participants from all districts expressed how no one from their community helps with the care of family members with mental health problems. Some participants mentioned that community members would rather stay away from the house all together, than come and keep her company. This was also exemplified in an interview with a woman in rural Palakkad who cares for her mother with psychosis.

‘They won’t help. They would say she is crazy mad and stays away’ (Caregiver 14, Rural Palakkad)

Caregiver 57, a male in his 60s, who cares for his wife, explained that ‘nobody has come personally to me or to my son to mock my wife’ but he goes on to explain that he thinks it is because ‘my wife is really nice to them’. Even so, participants expressed a suspicion that there remain a number of negative preconceptions around mental illness and participants felt as if they could not talk to anyone about their family members problems. Caregiver 57 went on to mention that ‘friends have become reluctant to come over’ due to his wife’s mental health problems and believed that they have not spoken to his wife after her diagnosis, because they assumed she was ‘violent’ and ‘nasty’.

Where stigma of this kind was mentioned, it was for the most part not seen to be improving. As one woman in Edappally indicated, there was still a degree of stigma attached to seeking help from a psychiatrist. She said she was even scared to go to Chottanikkara temple as it was attended by people who had mental illness were affected by spirits, which could have ‘affected her’, implying that she believed the symptoms might be contagious.

### Stigma and the family: Self-Stigma and collective responsibility

Across all four districts, there was an interesting theme which highlighted that many patients and caregivers had experienced a kind of self-stigma merely from caring for someone with a mental disorder or having someone with these difficulties in the family. Rather than self-stigma as it was originally characterised by Corrigan and Watson (2002) participants were more concerned about how this would reflect upon their families, rather than themselves. Patient 49, a woman from Chottankikkara poignantly explained how her family members would be getting judged because she had a mental disorder or was seeking help from a psychiatrist. Family support is one of the major pillars of community in India, which, when it works well, brings inclusion and a sense of belonging. If people feel that their problems are going to bring the disapprobation of their communities they will feel a sense of great shame, as mentioned above, and also a sense that it is their fault. Many participants felt that they would be excluded from society because they ‘consulted a psychiatrist’. Participant 50 a female patient from Chottanikkara, felt a sense of responsibility towards her son:

‘I asked him will you have to face any trouble in the future just because I am consulting a psychiatrist’ (Chottinikkara, Patient 50)

This was echoed across community members in Palakkad, many of whom expressed how many family members felt ‘shame’ and often ‘blamed the person with mental illness’ for having that condition. Participant 4 a female community member from urban Palakkad, who works with families who care for those with mental illness, said that family members express how they feel their relative with a mental disorder cannot go outside as it will affect the family’s social status. This suggests that rather than Corrigan and his colleagues’ very individualised notion of self-stigma, what is experienced in India not only involves the individual with problems but also concerns about how their mental ill-health reflects on the entire family.

In Edappally, there was greater acceptance among family members of the mental health problem and patients felt accepted and not ostracised, so there were fewer accounts of sufferers hiding their mental illness from their community:

‘My mom said like “don’t worry we are with you, and we will work on this”. And then she took me to psychiatrist because she somehow felt like may be this is…this is a little bit of anxiety or stress related issues so maybe we can go and consult a psychiatrist’ (Patient 58, Edapally)

Thus, it is possible in some cases for families to share the burden, not only of the symptoms but of possible disapprobation from the wider community too. This source of strength is a facet of the phenomena to which we shall return later.

## Discussion

The analysis of the interviews explored the types of stigma people described in their daily lives and attempted to relate it to the conceptions of stigma as these have been promoted in the literature of the Global North. Whilst there were many accounts of discrimination and exclusion, both in terms of lost opportunities and negative attitudes, there are some distinctive aspects worthy of note which have hitherto been neglected by scholars of stigma. The centrality of concerns about marriage and the eligibility of a person with a mental health problem as a marriage partner was a prominent feature of participants’ accounts. So much so that there were examples given of families who had successfully concealed these problems until after the marriage had been performed, lest it led to a refusal of the match by a prospective partner or their family.

A further finding of note was that there were no systematic differences across urban, rural, or tribal communities in the experience of stigma experienced by those with or associated with mental ill health. Nearly all participants from both rural and urban districts across Kerala spoke about both traditional methods of addressing mental health disorders – often their first resort – as well as seeking help from medical doctors and psychiatrists if the traditional or spiritual approach appeared to be ineffective. A sharp divide between rural and urban areas as found by, for example, Kishore et al. (2011) is far less easily detectable in the present study.

Despite India’s literacy rate improving, mental health literacy, at least in the sense intended by Jorm et al. (1997), seems to be less widespread. Many studies (e.g., Ahmed, 2019) have suggested that numerous anti-stigma campaigns and the promotion of a biomedical model have been unsuccessful due to the embedded stigma within communities. However, substituting a medical for a spiritual understanding of the problems experienced does not necessarily make people more humane, enlightened or compassionate. There is sometimes a fatalistic nihilism about telling someone they are suffering from a ‘brain disorder’, the practical consequences of which may be little different from a belief that they are possessed by an evil spirit. Perhaps campaigns could instead build upon the traditions of mutual support in Indian families and communities, promote understanding, kindness and compassion, or support for informal carers, rather than promoting a particular causal model or specific treatment options.

## Limitations

A limitation of the current study is that most participants were recruited at clinics. Despite efforts to say that all interviewers were researchers and not clinicians, participants were still likely to believe that the interviewers had something to do with the health care system. Therefore, they might be more likely to foreground medical and psychotherapeutic approaches rather than traditional healing practices.

As a single ‘snapshot’ of the situation in an individual interview may not be the most effective tool to examine the longer-term role of mental ill heath across the personal and family life course. The sensitivity of the subject matter may limit what people feel they can disclose to an interviewer. The precise details for the experiences detailed here may not be generalisable across India or other LMICs, but the overall point is that to understand stigma it is important to appreciate the local nuances of construct, rather than assume the concept will work the same way as it does in Europe and North America.

## Conclusion

In conclusion, in contrast to some previous research we did not observe any substantial difference between urban, rural/tribal areas. Patterns of prejudice and discrimination as well as negative expectations and typifications were common across all districts studied. Whilst there were parallels with notions of stigma originally introduced by Goffman and Corrigan and his colleagues there were distinctive aspects, such as the focus on marriage and concerns about the status of the family as a whole which had not so far been emphasised in European or north American scholarship. Interventions to improve the perceptions of mental ill health are still desirable but perhaps these would benefit from being focussed on particular aspects of a person’s life, for example improving marriage, understanding that people with mental health problems can function productively in many areas of life and visiting a psychiatrist does not mean a person is incompetent. These will need to be addressed in further research.
